# Base-Excision Repair Mutational Signature in Two Sebaceous Carcinomas of the Eyelid

**DOI:** 10.3390/genes14112055

**Published:** 2023-11-08

**Authors:** Eugenio Sangiorgi, Federico Giannuzzi, Clelia Molinario, Giulia Rapari, Melania Riccio, Giovanni Cuffaro, Federica Castri, Roberta Benvenuto, Maurizio Genuardi, Daniela Massi, Gustavo Savino

**Affiliations:** 1 Sezione di Medicina Genomica, Dipartimento di Scienze della Vita e Sanità Pubblica, Università Cattolica del Sacro Cuore, 00168 Roma, Italy; giuliarapari95@gmail.com (G.R.); melania.riccio01@icatt.it (M.R.); maurizio.genuardi@unicatt.it (M.G.); 2 Ocular Oncology Unit, Fondazione Policlinico Universitario A. Gemelli-IRCCS, Università Cattolica del Sacro Cuore, 00168 Roma, Italy; federico.giannuzzi@gmail.com (F.G.); giovanni.cuf@gmail.com (G.C.); gustavo.savino@unicatt.it (G.S.); 3 Division of Anatomic Pathology and Histology, Fondazione Policlinico Universitario A. Gemelli-IRCCS, Università Cattolica del Sacro Cuore, 00168 Roma, Italy; clelia.mol@hotmail.it (C.M.); federica.castri@policlinicogemelli.it (F.C.); roberta.benvenuto@policlinicogemelli.it (R.B.); 4 UOC Genetica Medica, Fondazione Policlinico Universitario A. Gemelli-IRCCS, 00168 Roma, Italy; 5 Section of Pathology, Department of Health Sciences, University of Florence, 50121 Florence, Italy; daniela.massi@unifi.it

**Keywords:** sebaceous gland carcinoma, mutational signature, Base-Excision Repair, whole exome sequencing

## Abstract

Personalized medicine aims to develop tailored treatments for individual patients based on specific mutations present in the affected organ. This approach has proven paramount in cancer treatment, as each tumor carries distinct driver mutations that respond to targeted drugs and, in some cases, may confer resistance to other therapies. Particularly for rare conditions, personalized medicine has the potential to revolutionize treatment strategies. Rare cancers often lack extensive datasets of molecular and pathological information, large-scale trials for novel therapies, and established treatment guidelines. Consequently, surgery is frequently the only viable option for many rare tumors, when feasible, as traditional multimodal approaches employed for more common cancers often play a limited role. Sebaceous carcinoma of the eyelid is an exceptionally rare cancer affecting the eye’s adnexal tissues, most frequently reported in Asia, but whose prevalence is significantly increasing even in Europe and the US. The sole established curative treatment is surgical excision, which can lead to significant disfigurement. In cases of metastatic sebaceous carcinoma, validated drug options are currently lacking. In this project, we set out to characterize the mutational landscape of two sebaceous carcinomas of the eyelid following surgical excision. Utilizing available bioinformatics tools, we demonstrated our ability to identify common features promptly and accurately in both tumors. These features included a Base-Excision Repair mutational signature, a notably high tumor mutational burden, and key driver mutations in somatic tissues. These findings had not been previously reported in similar studies. This report underscores how, in the case of rare tumors, it is possible to comprehensively characterize the mutational landscape of each individual case, potentially opening doors to targeted therapeutic options.

## 1. Introduction

Sebaceous gland carcinomas (SGC) are very rare malignant skin tumors typically categorized into two main groups based on their anatomical location. One group is located in the periocular region, while the second is typically found in sun-exposed areas of the head, neck, and trunk. While the cell of origin for periocular SGC is within the sebaceous gland of the upper eyelid, the origin of SGC present elsewhere on the body remains unknown [1,2].

In a comprehensive study of eyelid tumors, it was found that 85.7% were benign, 1.1% were premalignant, and 13.1% were malignant lesions. Of the malignant lesions, 60% originated from epidermal cells and 34.6% from adnexal cells. Specifically, 56.5% were basal cell carcinomas, 34.6% were sebaceous carcinomas, 3.8% were squamous cell carcinomas, and 1.7% were lymphoma or plasmacytoma [3]. The incidence varies significantly and has shown an increase over the years in different parts of the world. In the United States, SGC of the eyelid accounts for 4.7% of all eyelid cancers, while in India and China, it could represent up to 30% of all cases [4,5,6]. Onset typically occurs in adults over the age of 60, and extraocular SGC may be an indication of Muir-Torre syndrome, a phenotypic variant of Lynch syndrome characterized by a constitutional loss-of-function variant in a mismatch repair gene [7,8]. At the molecular level, Muir-Torre syndrome is associated with microsatellite instability, a specific molecular marker. Clinical trials have demonstrated the benefits of immunotherapies for systemic treatment in patients with Muir-Torre syndrome. Screening for this condition is generally recommended for patients with extraocular SGC under 50 years of age and those with a familial or personal history of Muir-Torre/Lynch syndrome-associated cancers.

The molecular profile of cutaneous malignant adnexal carcinomas has only been partially explored. Molecular analyses, including next-generation sequencing, have been conducted on small cohorts, examining targeted gene panels and/or whole exome sequencing [9,10,11,12,13]. Depending on the study, periocular SGC has been classified into three or two clinical entities. The three-class grouping was based on mutational signatures [9]. The first class includes SGC with microsatellite instability signatures 6 and 15, indicating a defect in the DNA mismatch repair system. The second class was associated with UV-related mutational signatures 7 and 11, as expected for tumors arising on the forehead. The third class, characterized by a low mutational burden and including all SGC of the eyelid, displayed predominantly signature 1, related to aging. The second classification defined only two subtypes [10]: the first associated with *TP53* and/or *RB1* variants along with *NOTCH1* mutations, and a second group lacking these mutations but showing integration and expression of high-risk human papillomavirus.

Two other commonly mutated genes in SGC are ZNF750 [14], particularly in the aforementioned pauci-mutational group, and PCDH15 [13], significantly associated with nodal and distant metastasis.

Periocular SGC displays particularly aggressive local behavior, and the recommended curative option is surgical excision, supplemented by topical mitomycin or cryotherapy for focally positive conjunctival margins. SGC with a stage of T2 or higher may be considered for sentinel lymph node dissection or biopsy [15,16,17]. Periocular SGC carries a high risk of local recurrence (8–11%), with 5.5% of cases resulting in distant metastases. Clear recommendations for systemic therapies, whether targeted or immunotherapies, for the management of metastatic disease are currently lacking. However, tumor profiling could potentially identify molecular targets amenable to targeted therapies.

Due to the low population prevalence, the diagnosis and therapeutic approaches for this group of tumors have not yet been well standardized. In this project, we aimed to characterize the mutational landscape of two sebaceous carcinomas of the eyelid following surgical excision. Utilizing available web bioinformatics tools, we identified that a large proportion of somatic mutations exhibited a Base-Excision Repair (BER) mutational signature. Additionally, they displayed a high tumor mutational burden (TMB), along with key driver mutations as evidenced by somatic substitutions, indels, and loss of heterozygosity (LOH) in several regions across the genome. These findings had not been previously reported in similar studies. This report underscores the paramount importance of conducting molecular profiling for rare cancers in order to provide the best possible curative options to patients.

## 2. Materials and Methods

### 2.1. Patients

The two patients, #1 and #2, were recruited from the Ocular Oncology Unit at the Fondazione Policlinico A. Gemelli, Rome. For each patient, their clinical history was evaluated, and standard tests and blood analyses were conducted during the pre-surgery visits. Following surgical excision, tumor staging and histopathological analyses were performed to confirm the diagnosis of undifferentiated sebaceous gland carcinoma of the eyelid.

During the enrollment process, we conducted a thorough examination of their family history, which did not raise any suspicion of Muir-Torre syndrome. Individuals #1 and #2 were 81 and 78 years old, respectively, at the time the eyelid lesion was detected. Patient #1 had no history of cancer in his family, but he was a smoker and had significant exposure to ultraviolet radiation. In the case of #2, her daughter had a history of acute myeloid leukemia. The study was approved by the Catholic University/Fondazione Policlinico Universitario A. Gemelli IRCCS Institutional Ethics Committee (protocol ID number: 5896).

Formalin-fixed, paraffin-embedded residual tumor and normal tissue were retrieved from the archive, and several 4-μm sections were obtained. These were stained with hematoxylin and eosin to identify healthy and tumor tissues, while the unstained sections were used for DNA extraction. An expert pathologist confirmed the predominance of tumor cells in the tumor sections (the fraction of cancer cells in the bioinformatics analysis was estimated to be 50% due to contamination of surrounding supportive tissue, blood cells, and blood vessels), while the normal tissue appeared devoid of cancer cells. DNA was extracted using standard protocols with the Qiagen QIAmp DNA FFPE tissue kit (Qiagen, Hilden, Germany). The purified DNA was quantified using standard spectrophotometric measurements.

### 2.2. Sequencing

Whole exome sequencing was performed by Dantelabs SRL (L’Aquila, Italy) using Illumina technology. Sequencing was conducted on a targeted library of 41.5 Mb of coding nucleotides. For the tumor samples of patient #1, sequencing generated 93 Gb of reads, while for patient #2, 98.5 Gb were generated. For the normal samples, 80 and 79 Gb of reads were generated, respectively.

### 2.3. Bioinformatics Analyses

Paired FASTQ files were processed on the Galaxy server [18] (https://usegalaxy.org accessed on 1 October 2023) following standard procedures. Reads were trimmed using Trimmomatic [19], then mapped with BWA-MEM [20], selecting high-quality, non-duplicated reads. All indel variants were left-aligned. To identify somatic variants, LOH events, and germline events, three different variant callers were used on the Galaxy platform: GATK4-Mutect2 [21], Strelka somatic [22], and Varscan somatic [23]. The main parameters for each software were the purity of the normal tissue set to 1, the purity of the tumor tissue set to 0.5, and the minimum base quality set to 28. For variant calling, the minimum coverage was set to 8 reads, with a minimum of 2 supporting reads, a variant allele frequency of 0.1, and the homozygous state for a tumor variant set to 0.75. For each software, we uploaded the BAM file properly filtered from the normal and tumor DNA. Each analysis from the three software tools yielded approximately the same number of somatic variants, and the remaining characterization was done exclusively with the variant caller Varscan somatic. All variants from Varscan somatic were classified as constitutional when present in both the normal and tumor DNA, somatic when present exclusively in the tumor DNA, and LOH events when the variant exhibited discordant zygosities between tumor and normal tissue DNA.

Variants passing the variant caller filter were then annotated using the wAnnovar [24] website (https://wannovar.wglab.org/ (accessed on 1 October 2023)). To identify constitutional variants potentially predisposing to cancer, all variants were selected to be exonic/splicing, no synonymous, and have a minor allele frequency below 1/1000 in the gnomAD database (https://gnomad.broadinstitute.org (accessed on 1 October 2023)).

Annotated somatic variants (tumor only) were characterized for their specific cancer molecular signature using three different web tools: Signal [25] (https://signal.mutationalsignatures.com (accessed on 1 October 2023)), Mutalisk [26] (http://mutalisk.org (accessed on 1 October 2023)), and SigProfiler assessment [27] (https://cancer.sanger.ac.uk/signatures/assignment (accessed on 1 October 2023)).

Signal analysis is based on the work of Degasperi, 2022 [25], where the primary cohort, referred to in Signal as GEL, is a collection of 15,838 whole-genome sequenced tumor samples made available as part of the Genomics England 100,000 Genomes Project (100 kGP). The signatures extracted from the GEL cohort were validated against two further cohorts: ICGC, 3001 primary WGS tumor samples from the International Cancer Genome Consortium, and Hartwig, 3417 metastatic WGS tumor samples from the Hartwig Medical Foundation.

Mutalisk is a free and public web service program that enables comprehensive analysis of somatic DNA mutations within genome regulatory elements and DNA sequence contexts. Systematic decomposition of mutational signatures was based on 30 known standard COSMIC mutational signatures [28].

SigProfiler Assessment provides the main output of the activity of each known mutational signature for each of the supplied samples. Signature activities correspond to the specific numbers of mutations from the original catalog caused by a particular mutational process [29].

To discriminate among the somatic mutations, identifying which ones could represent drivers, passengers, or non-affecting mutations, we used the Cancer Genome Interpreter [30] website (CGI; https://www.cancergenomeinterpreter.org (accessed on 1 October 2023)). This software is freely available and is designed to support the identification of tumor alterations that drive the disease and/or that may be therapeutically actionable. CGI relies on computational methods, in silico saturation mutagenesis of cancer genes (BoostDM and OncodriveMut), as well as knowledge collected across the public domain to annotate the alterations in a tumor according to several levels of evidence.

Constitutional, somatic, and LOH variants obtained from the different analyses were prioritized using the Varelect [31] software (https://varelect.genecards.org/ (accessed on 1 October 2023)) based on the following keywords: “cancer” OR “tumor suppressor gene” OR “BER” OR “hereditary cancer”.

### 2.4. Microsatellite Analysis

Microsatellite instability was evaluated using the PlentiPlex MSI Pentabase panel (Odense, Denmark), following the manufacturer’s recommendations. This assay contains the following five microsatellites: *BAT25*, *BAT26*, *MONO27*, *NR-22*, and *NR-24*.

## 3. Results

**Figure 1:**
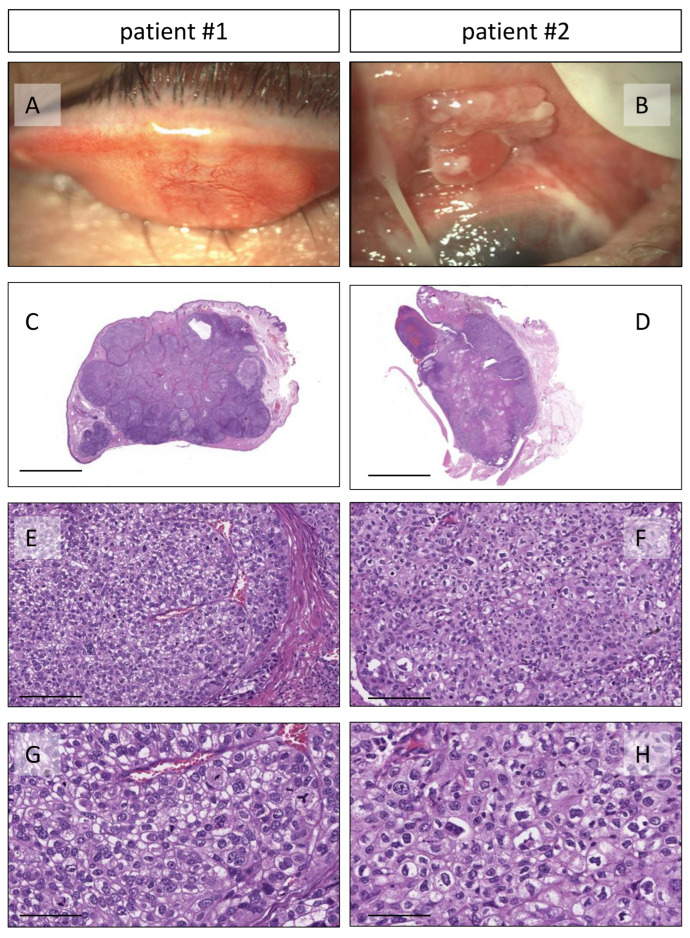
Representative images of the two SGCs of the eyelid. (**A**,**B**): Macroscopic photographs of the two tumors located on the upper eyelid. (**C**–**H**): Histopathological examination shows sheets of atypical sebocytes with clear and vacuolated (bubbly) cytoplasm and nuclear scalloping; haematoxylin and eosin stain of the two tumors at 1×, 20×, and 40× magnification; scale bars 500 µm, 25 µm, and 10 µm, respectively.

Patients #1 and #2 are 81 and 78 years old, respectively, with no significant comorbidities. They are in good health. Patient #1 was admitted to our Ocular Oncology Unit with conjunctival blushing and slight pain in the right upper eyelid. The biomicroscopic examination revealed a nodular infiltration of the right upper eyelid, measuring 15 mm in width and 13 mm in height, with a reddish appearance. An enlarged excisional biopsy was performed, followed by a histopathological examination. The upper eyelid was reconstructed using the Cutler-Beard technique. Tumor staging was determined as T2cN0M0. A histopathological diagnosis of poorly differentiated sebaceous carcinoma was made (Figure 1A,C,E,G).

Patient #2 was referred to us for a pedunculated, polylobular neoformation infiltrating the upper left eyelid. An incisional biopsy showed a poorly differentiated sebaceous carcinoma. Magnetic resonance imaging revealed invasion of orbital tissues with involvement of the superior and medial recti. The lymphoscintigraphy procedure detected two sentinel lymph nodes, one in the left preauricular region and the other posterior to the left angulomandibular area. An exenteration with lateral orbitotomy and parotid lymphadenectomy using a γ probe was performed. The histopathological examination confirmed a poorly differentiated invasive sebaceous carcinoma (Figure 1B,D,F,H), staged as T4N1M0. Both patients were free of recurrence at the 2-year follow-up.

DNA extracted from the tumor tissue and the normal surrounding tissue underwent exome sequencing. Sequencing of the tumor yielded much higher coverage compared to the normal tissue, likely due to a higher number of cells and an abundance of available tissue. In order to characterize germline, somatic, and LOH events, we compared the DNA extracted from the tumor and normal surrounding tissue using different variant caller software: GATK4 Mutect2, Strelka somatic, and Varscan somatic. The results from the three analyses returned approximately the same number of somatic variants. Here, we will discuss the results obtained from the Varscan somatic.

Varscan somatic reported approximately 1,000,000 variants for #1, but only 67,102 passed all the quality filters. Among these, 59,898 were germline, 2690 were somatic (tumor only), 4325 were LOH events, and the remaining 189 did not fall into any of the previous categories and represent probable artifacts in poorly covered regions. For #2, the corresponding numbers were as follows: out of 620,000 total variants, 56,588 passed all the filters. Among these, 48,626 were constitutional, 3714 were somatic (tumor only), 3863 were LOH events, and 385 did not belong to any of the previous groups.

The somatic mutations included single-base substitutions (SBS) and small indels. Patient #1 had 2518 SBS out of 2690, while #2 had 3515 SBS out of 3714; the rest of the variants were indels, representing respectively 6.3% and 5.3% of the total somatic events. We determined that for each tumor, the tumor mutation burden (TMB) was 84 mutations per megabase for #1 and 117 mutations for #2. Given the high mutation burden of each tumor, we decided to determine the molecular cancer signature of each tumor.

We used three different web platforms (Signal, Mutalisk, and SigProfiler Assignment) that provided overlapping results. From Signal, both tumors had their largest fractions of somatic SBS compatible with the SBS30 signature, which is related to BER deficiency, followed by the SBS1 signature related to aging. Patient #1 also had a 16% contribution from the SBS18 signature, again related to BER deficiency (Table 1).

Based on the molecular signature, the Signal platform identified that the topmost similar cancer samples in their database were Central Nervous System (CNS) and “bone and soft tissue” tumors (Table 2), despite our initial categorization as “head and neck cancer”.

From Mutalisk, patients #1 and #2 had their major fraction of variants attributed again to the SBS30 signature (BER deficiency) and to SBS23 (whose etiology remains unknown), with a smaller contribution from SBS7 (UV damage) and SBS1 (age-related) (Table 3).

SigProfiler Assignment returned for both samples an SBS30 dominant molecular signature.

The SBS30 signature has been previously associated with homozygous loss-of-function molecular lesions of the *NTHL1* tumor suppressor gene [32,33]. We searched for constitutional or somatic variants in this gene or in any of the genes related to the BER pathway, prioritizing genes with Varelect. We found that #2 was heterozygous for a germline stop codon variant in *NTHL1*. However, the variant was present in only 8 out of 42 reads. We also searched whether other regions of the *NTHL1* gene were involved in additional LOH events, but no suggestive alterations were present in the two tumors. We could not find additional mutational events (somatic or LOH) in both tumors in any of the known genes involved in the BER pathway. Since only the complete loss of *NTHL1* has been associated with an SBS30 signature, the cause of the SBS30 in these two tumors remains undetermined.

We then looked for additional constitutional variants in other tumor suppressor genes. Using Varelect, we prioritized all constitutional variants for both samples and, after filtering, we searched for pathogenic variants or variants of unknown significance (VUS) in well-established tumor suppressor genes. The only variant that emerged from this analysis was a heterozygous VUS in the *POLE* gene (NM_006231: c.74C>A; p.(Ala25Asp)) in #1. No constitutional pathogenic variants or VUS in any of the genes of the mismatch repair system were found.

We decided to investigate which somatic variants were driver or passenger variants. For this goal, all the somatic variants were uploaded to the CGI website and annotated. Patient #1 sample had driver mutations in *PTEN*, *TP53*, and *ZNF750*, along with seven other driver variants and 303 passenger mutations, while the remaining mutations were located in non-coding regions (Table 4). Patient #2 had an *RB1* variant along with 27 other driver mutations and 901 passenger mutations, and the remaining were classified as non-affecting mutations (Table 4). It was interesting to observe that both samples had a driver variant in the *ARID1B* gene. For #1, *PTEN* and *FAT1* were also involved in LOH events, suggesting that both genes potentially had both alleles inactivated, one by the somatic mutation and the other by a deletion. Patient #2 had several genes with somatic variants carrying LOH events (Table 4). We also wanted to check whether passenger variants had LOH events in the same gene. For this reason, we identified the top 10 genes carrying a somatic variant along with an LOH (Table 5) (Supplementary Table S1). Patient #2 carried a stop codon as a somatic event and an LOH of the *MSH6* gene, leading to the potential complete loss of the MSH6 protein.

Finally, we prioritized genes with just LOH events using Varelect, and as evident from Table 6, both patients had genes in all major pathways involved in tumorigenesis that were potentially deleted (Table 6 and Supplementary Table S2).

Considering the high TMB, presence of somatic variants and indels, and LOH in the mismatch repair pathway in #2, we decided to test the microsatellite instability in both tumor DNAs. Both cases resulted in low instability because only one microsatellite sequence each (out of five microsatellites) was unstable: *BAT25* for patient #1 and *BAT26* for patient #2.

## 4. Discussion

In this paper, we characterized two SGCs of the eyelid using exome sequencing. We discovered that both tumors exhibited a remarkably high TMB of 84 and 117 per Mb, with over 90% of the somatic mutations being single-base substitutions, carrying a BER deficiency cancer molecular signature. The remaining 5% and 6% comprised small indels. These findings are unexpected, as previous studies analyzing SGCs of the eyelid consistently revealed a low mutational load with a cancer signature that, while not highly significant due to the limited number of somatic mutations, was associated with aging and UV exposure.

Even in the case of other SGCs located in the head, neck, or trunk regions within the same studies, none displayed the SBS30 signature. Our exomes achieved high coverage (ranging from 150× to 300×), ensuring that all variants were covered by an average of 160 reads. Less than 10% had between 8 and 20 reads, thereby eliminating the possibility of the unreliable presence of these variants.

Furthermore, for the top 50 variants prioritized by Varelect, whether germline, somatic, or showing LOH, we directly confirmed their presence in the BAM file loaded onto the UCSC genome browser as a custom track. This step was taken to eliminate the chance that these variants might represent sequencing or mapping artifacts. The variant calling process was executed using three different software packages (Mutect2 (Galaxy Version 4.1.7.0+galaxy1), Strelka somatic (Galaxy Version 2.9.10+galaxy0), and Varscan somatic (Galaxy Version 2.4.3.6)). Additionally, the analysis of the molecular cancer signature was conducted using three distinct publicly available online tools (Signal, Mutalisk, and SigProfiler assessment), employing two different datasets of cancer genomic DNA. All these diverse analyses yielded converging and overlapping results for both samples.

The reasons why the two samples exhibit distinct results compared to the published findings remain unknown at this time. However, when examining driver mutations, our two samples presented *PTEN*, *TP53*, and *ZNF750* in one tumor and *RB1* in the other, confirming that these genes are primary somatic drivers for this type of cancer, as reported before. Moreover, we identified numerous other somatic events, including single-base substitutions, indels, and LOH, impacting all major pathways implicated in human tumorigenesis.

It is noteworthy that both tumors harbored driver mutations in *ARID1B*. While germline mutations of this gene are responsible for Coffin-Siris syndrome, a congenital neurodevelopmental disorder, the encoded protein is a component of the SWI/SNF chromatin remodeling complex [34]. This gene, along with other genes of the SWI/SNF complex, is frequently mutated in various cancers, often exhibiting high TMB and microsatellite instability [35]. It will be worth exploring in future studies of the SGC of the eyelid and the role of this protein.

The SBS30 BER deficiency signature was experimentally attributed to biallelic loss of function of the *NTHL1* tumor suppressor gene [32]. However, this signature is relatively uncommon among cancers, being observed in only a few breast cancers and one hepatocarcinoma. In these cases, loss of function variants in *NTHL1* were identified in only a small subset of samples, suggesting the possibility that other genes may lead to the same signature. Additionally, epigenetic silencing or other mutational events in *NTHL1*, not captured by whole exome or genome sequencing, could be present. In our patient #1, the variant in *NTHL1* was apparently mosaic in normal tissue and disappeared in the tumor DNA, without any evidence of a second mutational event in *NTHL1*. The other possible explanation is that this variant represents a sequencing artifact. For these reasons, its role in this patient as the cause of the BER deficiency signature remains to be determined. We then conducted a search for predisposing variants that could account for the high TMB, BER deficiency, and low microsatellite instability, but no pathogenic constitutional variants were detected. The only exception was a heterozygous variant of unknown significance in the *POLE* gene detected in #1. At present, it is challenging to reconcile this finding with the molecular and clinical phenotypes observed in this patient. Both patients were elderly, with a family history negative for hereditary cancer. Furthermore, the localization on the eyelid makes unlikely the presence of predisposing variants in the mismatch repair pathway or in other tumor suppressor genes. In fact, genes involved in the mismatch repair pathway were exclusively implicated at the somatic level with LOH, while the *MSH6* gene had a stop variant and a LOH. Biallelic somatic mutations of MMR genes have been observed in different types of cancers in the spectrum of Lynch-Muir-Torre syndrome [36,37]. These phenocopies usually have clinical-pathological characteristics similar to those of their hereditary counterparts. Therefore, it is likely that in patient #2, somatic inactivation of *MSH6* could underlie the high TMB and low microsatellite instability, although additional undetected events could be involved in causing a high mutation rate.

Rare genetic diseases, as well as rare cancers, often face neglect from major funding agencies and collective scientific efforts. In most cases, the molecular pathogenesis is yet undefined, and large-scale clinical trials cannot be performed. However, the increasing availability of technological advancements now allows us to apply the same tools utilized for common cancers to study very rare ones. SGC of the eyelid is notably absent from the most common cancer genome datasets, such as COSMIC and others. Due to their rarity, therapeutic options are primarily based on case reports or very small series. Nevertheless, this rarity allows for a more personalized approach, as demonstrated by some successful gene therapy cases [38]. This study aimed to serve as a proof of concept to demonstrate that within the typical timeframe of a diagnostic procedure, by utilizing publicly available bioinformatics tools, we can comprehensively characterize two rare tumors. This not only expands our scientific understanding of these tumors but also provides a series of potential actionable mutations that can be targeted by therapies already in use for other cancers.

It may be challenging to envision a large-scale clinical trial for systemic therapies for SGCs of the eyelid. However, the modern targeted pharmacological approach is becoming increasingly agnostic to histology and is primarily driven by the presence of specific gene variants. This approach will likely be applied to rare cancers once the key driver variants are identified.

## Figures and Tables

**Table 1:** Results of the mutational cancer signature contribution obtained from Signal online tools are presented.

	Mutational Signatures from Signal			
Patient #1	SBS30 54% BER deficiency	SBS1 28% Deamination	SBS18 16% BER deficiency	Unassigned 1%
Patient #2	SBS30 68% BER deficiency	SBS1 30% Deamination		Unassigned 2%

**Table 2:** Results of the comparison between the mutational cancer signature contribution obtained from Signal online tools and the top 5 most similar cancer samples.

	Top 5 Most Similar Cancer Samples from Signal				
Patient #1	GEL-2944131-11 CNS 0.877	GEL-2478942-11 CNS 0.797	GEL-2808113-11 Bone and soft tissues 0.761	DO52610 Bone and soft tissues 0.748	DO52610 NET 0.74
Patient #2	GEL-2944131-11 CNS 0.917	GEL-2478942-11 CNS 0.856	GEL-2808113-11 Bone and soft tissues 0.827	DO52610 Bone and soft tissues 0.807	HMF0030688 Bone and soft tissues 0.796

**Table 3:** Results of the mutational cancer signature obtained from Mutalisk online tools are presented.

Mutational Signatures from Mutalisk Patient #1			
Signatures	Probabilities	Cosine	BIC
23 30 1 7 22 18	0.27861 0.26564 0.17885 0.13563 0.07967 0.06159	0.82600	17,428.410
30 23 1 7 11 22 18	0.24343 0.23671 0.18389 0.10866 0.08411 0.08106 0.06216	0.82700	17,435.600
30 23 1 7 22	0.30204 0.27441 0.20025 0.13004 0.09325	0.82300	17,654.570
30 23 1 7	0.40833 0.26234 0.22351 0.10581	0.81500	17,970.830
30 23 1	0.54416 0.25502 0.20081	0.80800	18,044.040
1	1.00000	0.74800	19,951.940
30 23	0.75283 0.24717	0.78800	21,139.930
**Mutational signatures from Mutalisk Patient #2**			
**Signatures**	**Probabilities**	**Cosine**	**BIC**
30 23 1 7 11 22 4	0.30286 0.26799 0.20011 0.11531 0.07099 0.03274 0.01000	0.90100	23,356.170
30 23 1 7 11 22	0.30818 0.26830 0.20242 0.11439 0.07076 0.03594	0.90100	23,392.800
30 23 1 7 22	0.32650 0.30361 0.19804 0.13716 0.03469	0.90000	23,397.800
30 23 1 7	0.36591 0.29917 0.20674 0.12819	0.89900	23,550.040
30 23 1	0.53017 0.29046 0.17937	0.89100	23,718.120
30 23	0.71638 0.28362	0.87200	26,641.600
19	1.0000	0.82400	26,767.810

**Table 4:** Somatic driver variants identified through the analysis of the CGI.

Patient	Mutation	Gene	Protein Change	CGI-Oncogenic Summary	Consequence	LOH
#1	chr10:89717672 C>T	PTEN	R233X	oncogenic (predicted and annotated)	stop_gained	YES
#1	chr17:7578419 C>A	TP53	E171X	oncogenic (predicted)	stop_gained	NO
#1	chr17:80790252-80790252 ATTT>-	ZNF750	KC26-27X	oncogenic (predicted)	frameshift_variant	NO
#1	chr22:37708082 C>T	CYTH4	P327S	oncogenic (predicted)	missense_variant	NO
#1	chr2:141116501 G>A	LRP1B	P3716S	oncogenic (predicted)	missense_variant	NO
#1	chr3:52610685 G>A	PBRM1	A1188V	oncogenic (predicted)	missense_variant	NO
#1	chr4:54319218 C>T	FIP1L1	R473C	oncogenic (predicted)	missense_variant	NO
#1	chr4:187557927 G>A	FAT1	R1262X	oncogenic (predicted)	stop_gained	YES
#1	chr6:157502150 C>G	ARID1B	Y1184X	oncogenic (predicted)	stop_gained	NO
#1	chr7:143098557 C>T	EPHA1	E98K	oncogenic (predicted)	missense_variant	NO
#2	chr13:48951171 G>T	RB1	--	oncogenic (predicted and annotated)	splice_donor_variant	NO
#2	chr12:992225 G>A	WNK1	M1576I	oncogenic (predicted)	missense_variant	NO
#2	chr12:56482607 C>A	ERBB3	T355N	oncogenic (predicted)	missense_variant	NO
#2	chr13:35734171 T>A	NBEA	--	oncogenic (predicted)	splice_donor_variant	YES
#2	chr13:35756496 G>T	NBEA	--	oncogenic (predicted)	splice_acceptor_variant	YES
#2	chr14:21875095 G>A	CHD8	R943C	oncogenic (predicted)	missense_variant	NO
#2	chr15:28231765 C>T	OCA2	E403K	oncogenic (predicted)	missense_variant	YXES
#2	chr16:58585089 C>T	CNOT1	E1097K	oncogenic (predicted)	missense_variant	NO
#2	chr17:1561923 G>A	PRPF8	P1758L	oncogenic (predicted)	missense_variant	YES
#2	chr17:7800428 C>T	CHD3	R638X	oncogenic (predicted)	stop_gained	YES
#2	chr17:8050603 C>T	PER1	G532R	oncogenic (predicted)	missense_variant	NO
#2	chr17:56438147-56438147 C>-	RNF43	G282X	oncogenic (predicted)	frameshift_variant	NO
#2	chr17:62496853 G>A	DDX5	P419S	oncogenic (predicted)	missense_variant	YES
#2	chr18:50912508 G>A	DCC	D819N	oncogenic (predicted)	missense_variant	YES
#2	chr19:9084065 C>A	MUC16	G2584X	oncogenic (predicted)	stop_gained	YES
#2	chr1:21190980 C>T	EIF4G3	E1132K	oncogenic (predicted)	missense_variant	NO
#2	chr1:155920771 C>T	ARHGEF2	R994H	oncogenic (predicted)	missense_variant	NO
#2	chr1:197091163 G>A	ASPM	T1251I	oncogenic (predicted)	missense_variant	NO
#2	chr22:20074748 C>T	DGCR8	R262X	oncogenic (predicted)	stop_gained	YES
#2	chr3:13672957 G>A	FBLN2	D1072N	oncogenic (predicted)	missense_variant	NO
#2	chr3:71050163 C>G	FOXP1	C341S	oncogenic (predicted)	missense_variant	NO
#2	chr3:185146526 C>T	MAP3K13	R53X	oncogenic (predicted)	stop_gained	NO
#2	chr6:94068121 G>A	EPHA7	R281C	oncogenic (predicted)	missense_variant	YES
#2	chr6:157517340 C>A	ARID1B	P1425T	oncogenic (predicted)	missense_variant	YES
#2	chr8:31024573 C>T	WRN	P1340S	oncogenic (predicted)	missense_variant	YES
#2	chr8:103340036 C>T	UBR5	R472Q	oncogenic (predicted)	missense_variant	NO
#2	chr9:8633420 T>A	PTPRD	R83S	oncogenic (predicted)	missense_variant	NO
#2	chrX:44923062 G>A	KDM6A	Q693	oncogenic (predicted)	splice_region_variant	YES

There are 10 driver variants for patient #1 and 27 for patient #2. In the last column (LOH), it is indicated whether the gene also experienced a LOH event elsewhere in the tumor. The presence of LOH in the same gene suggests that both alleles could potentially be lost.

**Table 5:** List of the top 20 genes exhibiting LOH in each patient, prioritized using Varelect.

Patient	Top 20 Genes with LOH	Gene Name
#1	BRCA2	BRCA2 DNA Repair-Associated
#1	BRIP1	BRCA1 Interacting Helicase 1
#1	NF1	Neurofibromin 1
#1	BARD1	BRCA1 Associated RING Domain 1
#1	PTEN	Phosphatase and Tensin Homolog
#1	AXIN2	Axin 2
#1	STK11	Serine/Threonine Kinase 11
#1	RET	Ret Proto-Oncogene
#1	RAD51C	RAD51 Paralog C
#1	KIT	KIT Proto-Oncogene, Receptor Tyrosine Kinase
#1	BMPR1A	Bone Morphogenetic Protein Receptor Type 1A
#1	FLCN	Folliculin
#1	ERBB2	Erb-B2 Receptor Tyrosine Kinase 2
#1	FANCC	FA Complementation Group C
#1	SUFU	SUFU Negative Regulator of Hedgehog Signaling
#1	GALNT12	Polypeptide N-Acetylgalactosaminyltransferase 12
#1	PRKAR1A	Protein Kinase CAMP-Dependent Type I Regulatory Subunit α
#1	STAT3	Signal Transducer and Activator of Transcription 3
#1	ERBB3	Erb-B2 Receptor Tyrosine Kinase 3
#1	LRRC56	Leucine Rich Repeat Containing 56
#2	BRCA1	BRCA1 DNA Repair Associated
#2	ATM	ATM Serine/Threonine Kinase
#2	PALB2	Partner and Localizer of BRCA2
#2	MSH6	MutS Homolog 6
#2	MSH2	MutS Homolog 2
#2	CDH1	Cadherin 1
#2	BRIP1	BRCA1 Interacting Helicase 1
#2	PMS2	PMS1 Homolog 2, Mismatch Repair System Component
#2	POLE	DNA Polymerase Epsilon, Catalytic Subunit
#2	NF1	Neurofibromin 1
#2	TSC2	TSC Complex Subunit 2
#2	ALK	ALK Receptor Tyrosine Kinase
#2	DICER1	Dicer 1, Ribonuclease III
#2	RET	Ret Proto-Oncogene
#2	NTHL1	Nth Like DNA Glycosylase 1
#2	MLH3	MutL Homolog 3
#2	ERBB2	Erb-B2 Receptor Tyrosine Kinase 2
#2	PALLD	Palladin, Cytoskeletal-Associated Protein
#2	WT1	WT1 Transcription Factor
#2	AOPEP	Aminopeptidase O (Putative)

**Table 6:** List for each patient of the top 10 passenger gene variants identified from CGI, which also exhibit a LOH event elsewhere in the same gene. This suggests that potentially both alleles are non-functional.

Patient	Gene	Variant	Consequence
#1	ALDH1A1	chr9:75543807 C>T	splice_donor_variant
#1	ABCA1	chr2:215884469 T>A; p.(Ser447Cys)	missense_variant
#1	CAGE1	chr6:7370306 C>A	splice_region_variant
#1	RUBCNL	chr13:46918890 C>T; p.(Cys621Tyr)	missense_variant
#1	COL4A2	chr13:111156539 G>T; p.(Gly1444Cys)	missense_variant
#1	PLCE1	chr10:96081817 G>A	splice_donor_variant
#1	SYNE1	chr6:152683380 C>T; p.(Met3408Ile)	missense_variant
#1	MADCAM1	chr19:501786 C>A; p.(Pro286Gln)	missense_variant
#1	NET1	chr10:5498201 G>A; p.(Gly450Asp)	missense_variant
#1	HSPG2	chr1:22170691 C>T; p.(Ala2856Thr)	missense_variant
#2	MSH6	chr2:48027853 C>T; p.(Arg911ter)	stop_gained
#2	ERBB2	chr17:37881572 C>T	splice_region_variant
#2	PALLD	chr4:169825046 G>A; p.(Ala871Thr)	missense_variant
#2	FANCD2	chr3:10106408 C>T	splice_region_variant
#2	KMT2C	chr7:151970859 C>T; p.(Gly315Ser)	missense_variant
#2	TTN	chr2:179584566 G>A	splice_region_variant
#2	FASN	chr17:80041523 G>A	splice_region_variant
#2	LIG1	chr19:48665601 A>T; p.(Phe9Ile)	missense_variant
#2	DSPP	chr4:88533649 G>A; p.(Gly104Glu)	missense_variant
#2	ITGA6	chr2:173330387 C>G; p.(Asn101Lys)	missense_variant

## Supplementary Materials

The following supporting information can be downloaded at: https://www.mdpi.com/article/10.3390/genes14112055/s1. Table S1: Somatic passenger variants; Table S2: Mutations with LOH events.
